# Simulating the Impact of Long-Term Care Prevention Among Older Japanese People on Healthcare Costs From 2020 to 2040 Using System Dynamics Modeling

**DOI:** 10.3389/fpubh.2020.592471

**Published:** 2020-12-14

**Authors:** Nobuo Nishi, Nayu Ikeda, Takehiro Sugiyama, Kayo Kurotani, Motohiko Miyachi

**Affiliations:** ^1^International Center for Nutrition and Information, National Institute of Health and Nutrition, National Institutes of Biomedical Innovation, Health and Nutrition, Tokyo, Japan; ^2^Institute for Global Health Policy Research, Bureau of International Health Cooperation, National Center for Global Health and Medicine, Tokyo, Japan; ^3^Department of Health Services Research, Faculty of Medicine, University of Tsukuba, Tsukuba, Japan; ^4^Department of Nutritional Epidemiology and Shokuiku, National Institute of Health and Nutrition, National Institutes of Biomedical Innovation, Health and Nutrition, Tokyo, Japan; ^5^Faculty of Life and Environmental Sciences, Showa Women's University, Tokyo, Japan; ^6^Department of Physical Activity Research, National Institute of Health and Nutrition, National Institutes of Biomedical Innovation, Health and Nutrition, Tokyo, Japan

**Keywords:** Japan, medical expenditure, long-term care expenditure, simulation model, system dynamics

## Abstract

**Objectives:** This study examined how healthcare costs might change by reducing long-term care needs among older Japanese people.

**Methods:** A simulation model was constructed comprising two aging chains for independent and dependent people aged ≥65 years by sex. Changes in the base run from 2020 to 2040 were compared with those in two hypothetical scenarios: a 2% annual reduction in death rates (S1), and S1 plus a 2% annual reduction in the proportion of dependent people aged 65 years and in transition rates from the independent to dependent state for people aged ≥65 years (S2).

**Results:** In the base run, the population increased by 13.0% for men and 11.3% for women, and the proportion of dependent people increased by 4.6% for men but decreased by 13.4% for women. The sum of medical and long-term care expenditure increased in the base run, S1, and S2 by 8.2, 27.4, and 16.4%, respectively, for men and women combined.

**Conclusions:** Healthcare costs will increase as death rates fall, but the increase will be attenuated if the proportion of dependent people decreases.

## Introduction

With a low mortality and a low birth rate, the Japanese population has been aging recently. In 2017, life expectancy was 81.09 years for men and 87.26 years for women (1), and the fertility rate was 1.43 (2). In 2018, the population aged 65 years or older accounted for 28.1% of the total population (3) which is the highest in the world. National medical expenditure has increased over time, reaching 7.8% of gross domestic product in 2016 (4). Thus, Japan is facing a growing burden in terms of healthcare costs that no other countries have ever encountered.

To control the rise in healthcare costs for older people, it is important that they remain in good health during the extra years of life gained through improved life expectancy. The extension of healthy life expectancy is one of the two main goals of Health Japan 21 (the second term), the government's national health promotion plan from 2013 to 2022 (5). Healthy life expectancy will increase through a decrease in the proportion of the population requiring medical and long-term care, which will decrease healthcare costs in the short term. However, the increase in healthy life expectancy will also change the age structure of the population by increasing the proportion of older people, and thus healthcare costs may increase in the long term.

Economics scholars have previously projected future healthcare costs for Japan (6, 7). However, they relied on projected changes in the general Japanese population and did not consider changes in the proportion of the population that is unhealthy. Fukawa (8) used a dynamic microsimulation model to show that total health and long-term care expenditure for older people would increase until 2050, and that a reduction in the prevalence of long-term care needs would significantly mitigate the increase in long-term care expenditure. However, their model was based on projections at the household level, and they did not implement a projection by sex.

Population aging is an important issue both in developed and developing countries. An accelerated population aging may cause a serious economic problem in the near future in low- and middle-income countries and emerging countries like BRICs (Brazil, Russia, India, and China), where medical and long-term care systems are not so much established as those in Japan (9, 10). A case report from Japan may help those countries to develop their own strategies for population aging. Therefore, the current study aimed to project changes in healthcare costs through prevention of mortality and long-term care needs among the older Japanese population. Simulation models using system dynamics were developed separately by sex to consider different rates of mortality and disability requiring long-term care.

## Methods

### Model Structure

**Figure 1:**
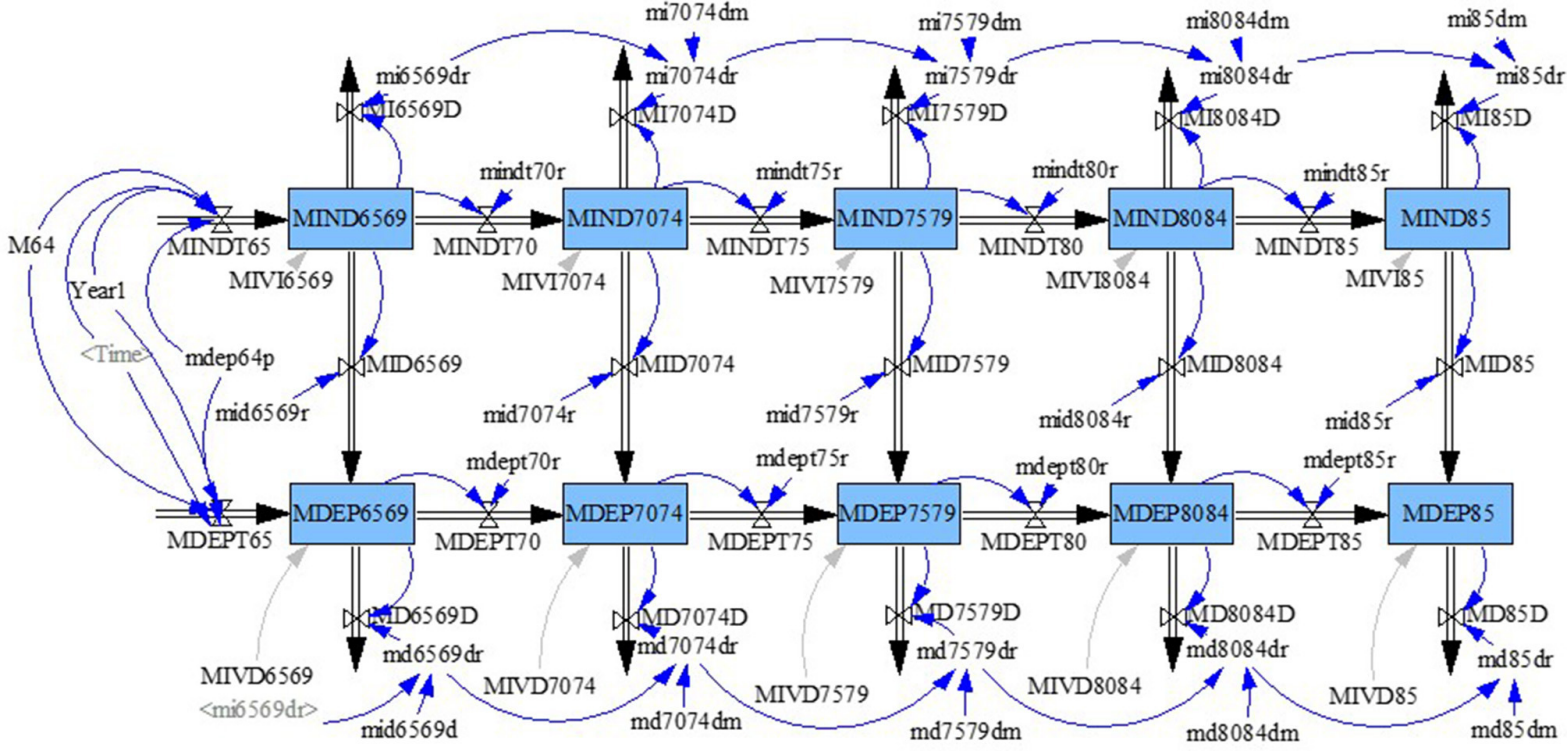
Model of transitions between independent and dependent states in men aged 65 years or older.

We followed the long-term care insurance system in Japan and defined dependent people as those in need of at least supervision for dressing and assistance for all or part of toileting and bathing who were certified as Level 2 to Level 5 (Level 5 is the highest level of care). A system dynamics model (11) was constructed comprising two aging chains for independent and dependent populations aged ≥65 years from 2010 to 2040 separated by sex to reflect differences in death rates and proportions of dependent people by sex and age group. The stock-flow diagram for men is shown in Figure 1. The five stocks in each aging chain represent 5-years age groups (65–69, 70–74, 75–79, 80–84, and ≥85 years). For example, MIND6569 and MDEP6569 indicate men aged 65–69 years who were independent and dependent, respectively. Each stock had outflows of deaths (e.g., MI6569D and MD6569D). Stocks were connected within age groups by transition flows from the independent aging chain to the dependent aging chain (e.g., MID6569).

Rate parameters were assigned to each of these flows (e.g., mi6569dr, md6569dr, and mid6569r). The numbers of people exiting from a stock or moving between stocks were calculated as the products of parameters (constant) and the number of people in the stocks from which the flows originated. Parameters were set on the assumption that the death rate increases with age and is higher in dependent people than in independent people within age groups.

People aged 64 years entered as inflows into the stocks of people aged 65–69 years in independent and dependent aging chains (MINDT65 and MDEPT65 in Figure 1). The number of people aged 64 each year (M64) was divided into independent and dependent aging chains with their respective parameters (1-mdep64p, mdep64p; constant). The number of people aged 64 was obtained from those in the previous years for 2010–2017 and the number of people reaching 64 years of age in 2017 was used for 2018–2040. For example, the numbers of people aged 63 and 62 in 2017 were used as the numbers of people aged 64 in 2018 and 2019, respectively. For simplicity, population change under 65 years of age was not considered in the analysis of future years.

### Medical and Long-Term Care Expenditure

Medical care expenditure was calculated separately for independent and dependent people as the product of per capita medical care expenditure (constant) and the number of people by sex and age group. It was assumed that per capita medical care expenditure for dependent people was higher than that for independent people in the same sex and age groups. Long-term care expenditure was calculated only for dependent people as the product of per capita long-term care expenditure (constant) and the number of dependent people by sex and age group.

### Reference Data

Reference data by sex and age group were obtained from national statistics reports (see Table 1). Regarding reference data for long-term care, only monthly statistics were available from a report on long-term care insurance payments (12). Therefore, the numbers of long-term care recipients were calculated as the average of the monthly numbers of recipients, and annual long-term care expenditure was obtained by summing the monthly expenditures. Data on long-term care expenditure were available only by age group, and thus data by sex and age group were calculated on the assumption that the male/female ratio in relation to long-term care expenditure was equivalent to that of the number of long-term care recipients in each age group.

**Table 1:** Statistics for reference data for the period 2010–2017 by sex and age group.

**Variable**	**Statistics**
Population	Population Estimates (yearly)
Number of deaths	Vital Statistics (yearly)
Medical care expenditure	Estimates of National Medical Care Expenditure (yearly)
Number of long-term care recipients^a^	Report of Long-term Care Benefit Expenditures (monthly)
Long-term care expenditure^b^	Report of Long-term Care Benefit Expenditures (monthly)

a *Average of monthly numbers of recipients*.

b *Sum of monthly expenditures over 12 months. Data on long-term care expenditure were available only by age group, and data by sex and age group were calculated on the assumption that the male/female ratio in relation to long-term care expenditure was equivalent to that of the number of long-term care recipients in each age group*.

### Optimization

Rate parameters for deaths and transition from the independent state to the dependent state, as well as per capita medical and long-term care expenditure, were calibrated to fit all of the abovementioned reference data by sex and age group. The inverse of the standard errors of the reference data were used as weights in the calibration.

### Validation of the Model

The validity of the model was examined in the following three ways. First, the model fit was checked for each stock based on sex and age group. Second, the parameters were checked in relation to the assumptions about death rates and per capita medical and long-term care expenditure. Third, the parameters were compared between independent and dependent people and between men and women to confirm that there were no extreme differences.

### Scenarios

On the basis of the optimization, the base run was obtained through 2040 using reference data trends between 2010 and 2017. Additionally, two hypothetical scenarios were set to simulate the effects of changes in death rates and rates of transition from the independent state to the dependent state from 2020 to 2040. A lookup variable was added to change the model parameters for the scenarios. An annual reduction of 2% (33% reduction in total by 2040) was used in the scenarios to show a clear contrast to the base run:

Scenario 0: base run
Scenario 1: 2% annual reduction in death rates
Scenario 2: Scenario 1 plus a 2% annual reduction in the proportion of dependent people at age 65 and in transition rates from the independent state to the dependent state for people aged ≥65 years.

## Results

### Validation of the Model

The mean absolute percentage error (MAPE) (11) ranged from 0.8 to 10.2 (average 3.4) for men and from 0.8 to 40.7 (average 6.9) for women.

### Changes in Population, Proportion of Dependent People, and Death Rates

Simulated values for the population aged 65 years and older, the proportion of dependent people, and death rates in 2010, 2020, and 2040 are shown by sex in Table 2. The population, proportion of dependent people, and death rates all increased from 2010 to 2020 for both men and women. The population was projected to increase by 13.0% for men and 11.3% for women from 2020 to 2040 in the base run, and was also projected to increase in S1 and S2 by 20.2 and 25.5%, respectively, for men and by 20.1 and 23.2%, respectively, for women. From 2020 to 2040, the proportion of dependent people was projected to increase by 4.6% for men but decrease by 13.4% for women in the base run, to increase by 36.5% for men and by 3.3% for women in S1, and to increase by 2.4% for men but to decrease by 23.7% for women in S2. Over the same period, the death rate was projected to increase by 5.5% for men but to decrease by 2.8% for women in the base run, to decrease by 11.7% for men and by 25.9% for women in S1, and to decrease by 25.2% for men and by 34.8% for women in S2.

**Table 2:** Simulated values for the population, dependent population, and deaths by age and sex and changes from 2020 to 2040 by scenario for people aged 65 years or older.

	**Simulated values**					**Change from 2020 to 2040**		
	**2010**	**2020**	**2040**					
			**Scenario 0**	**Scenario 1**	**Scenario 2**	**Scenario 0 (%)**	**Scenario 1 (%)**	**Scenario 2 (%)**
**Male**								
Population (1000)	12449.3	15550.2	17568.4	18688.4	19523.2	13.0	20.2	25.5
Number of dependent people (1000)	743.7	992.0	1172.8	1627.1	1275.7	18.2	64.0	28.6
Proportion of dependent people (%)	6.0	6.4	6.7	8.7	6.5	4.6	36.5	2.4
Number of deaths (1,000/year)	496.5	674.4	804.2	715.3	633.2	19.2	6.1	−6.1
Death rate (1,000/year)	39.9	43.4	45.8	38.3	32.4	5.5	−11.7	−25.2
**Female**								
Population (1000)	14557.8	17829.2	19846.1	21421.4	21973.5	11.3	20.1	23.2
Number of dependent people (1000)	1672.4	2215.7	2135.1	2749.5	2082.5	−3.6	24.1	−6.0
Proportion of dependent people (%)	11.5	12.4	10.8	12.8	9.5	−13.4	3.3	−23.7
Number of deaths (1,000/year)	492.9	694.4	751.0	618.1	557.7	8.2	−11.0	−19.7
Death rate (1,000/year)	33.9	38.9	37.8	28.9	25.4	−2.8	−25.9	−34.8

*Scenario 0: base run; Scenario 1: 2% annual reduction in death rates; Scenario 2: Scenario 1 plus 2% annual reduction in the proportion of dependent people aged 65 years and in the transition rates from the independent state to the dependent state for people aged ≥65 years*.

### Changes in Medical and Long-Term Care Expenditure

**Figure 2:**
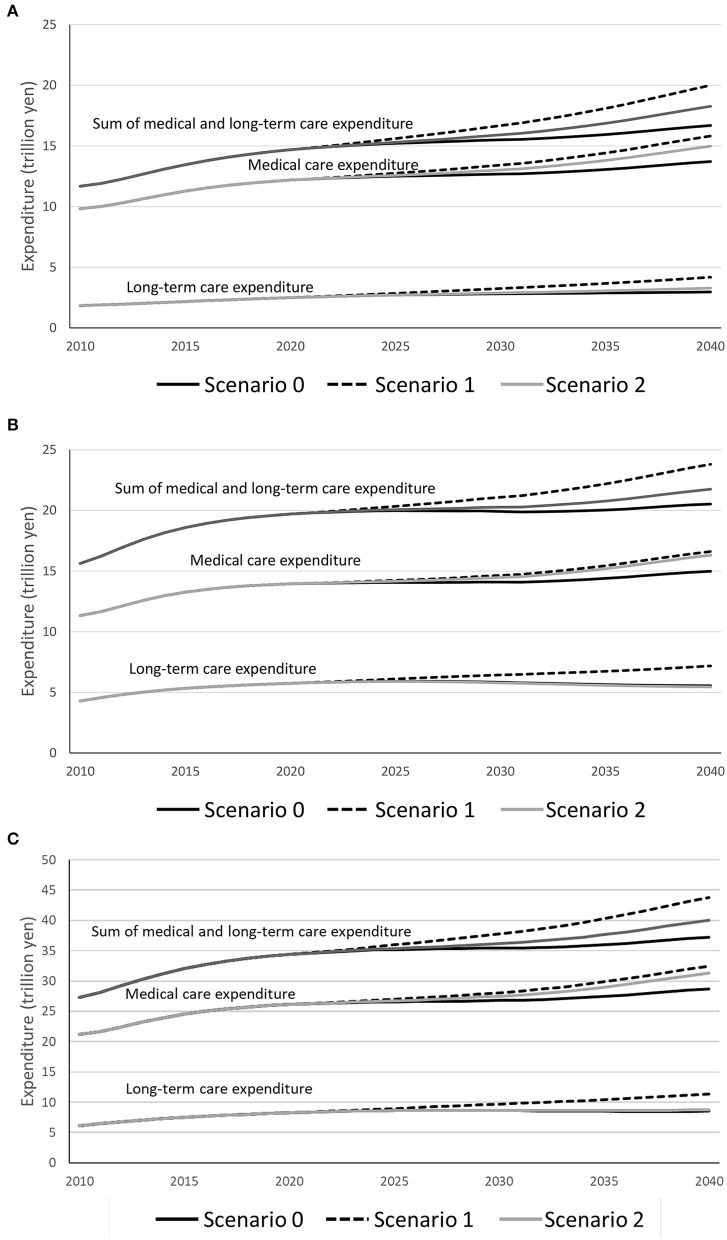
Simulated changes in medical care expenditure, long-term care expenditure, and the sum of medical and long-term care expenditure by scenario for **(A)** men, **(B)** women, and **(C)** men and women combined.

Simulated changes in medical care expenditure, long-term care expenditure, and the sum of medical and long-term care expenditure are shown by scenario for men (see Figure 2A), women (see Figure 2B), and men and women combined (see Figure 2C). For men, medical care expenditure was projected to increase by 12.5% in S0 (13.7 trillion yen in 2040), 29.8% in S1, and 22.9% in S2, long-term care expenditure was projected to increase by 19.0% in S0 (3.0 trillion yen in 2040), 67.6% in S1, and 31.8% in S2, and the sum of medical and long-term care expenditure was projected to increase by 13.6% in S0 (16.7 trillion yen in 2040), 36.2% in S1, and 24.4% in S2 from 2020 to 2040. For women, medical care expenditure was projected to increase by 7.4% in S0 (15.0 trillion yen), 19.1% in S1, and 16.9% in S2, long-term care expenditure was projected to decrease by 3.6% in S0 (5.5 trillion yen in 2040), to increase by 25.0% in S1, and to decrease by 5.3% in S2, and the sum of medical and long-term care expenditure was projected to increase by 4.2% in S0 (20.5 trillion yen in 2040), 20.8% in S1, and 10.4% in S2 from 2020 to 2040. For men and women combined, medical care expenditure was projected to increase by 9.8% in S0 (28.7 trillion yen in 2040), 24.1% in S1, and 19.7% in S2, long-term care expenditure was projected to increase by 3.3% in S0 (8.5 trillion yen in 2040), 37.9% in S1, and 6.0% in S2, and the sum of medical and long-term care expenditure was projected to increase by 8.2% in S0 (37.2 trillion yen in 2040), 27.4% in S1, and 16.4% in S2 from 2020 to 2040. By 2040, the sum of medical and long-term care expenditure for men and women combined was projected to be 37.2 trillion yen in S0, 43.8 trillion yen in S1, and 40.3 trillion yen in S2.

## Discussion

Some simulation studies have been conducted on population aging in Japan. Parsons and Gilmour (13) used stock flow population models to show that policy responses based on fertility and migration will not be able to reverse Japan's aging population within the next few decades. Chen et al. (14) adapted a demographic and economic state-transition micro-simulation model named the Future Elderly Model to Japan, and found that the impact of population aging exceeds the effect of age-specific morbidity on increasing disability. However, none of them estimated the change of medical and long-term care expenditure. Thus, to the best of our knowledge, this is the first simulation study to examine projected changes in medical and long-term care expenditure resulting from improvements in mortality and long-term care needs among older Japanese people. The results suggest that a reduction in death rates alone would increase medical and long-term care expenditure as the population increased, but that the increase would be suppressed if the transition rate from the independent state to the dependent state was reduced. The simulation was conducted by sex and the sum of medical and long-term care expenditure was projected to be higher in women than in men.

On the basis of the scenario of the extension of healthy life expectancy in the Health Japan 21 strategy (the second term), Tomata et al. (15) estimated the cost savings in relation to long-term care and medical care using population projections for Japan. They showed that ~2.5–5.3 trillion yen would be saved in long-term care and medical care costs by achieving the target stated in the scenario. However, their estimation was simply based on a reduction in the number of disability (unhealthy) cases, and long-term care and medical care costs in the future based on the survival of those who did not become unhealthy was not considered. The present study overcomes this limitation by using a simulation model to estimate the cost of long-term care and medical care in the future.

Fukawa (8) projected the increase in total health and long-term care expenditure until 2050 using a dynamic microsimulation model. Several scenarios were considered by changing the take-up rate (proportion of recipients) of long-term care. The main difference between the simulation model used in the present study and Fukawa's model is that the model used in the present study is based on individuals instead of households, and projected changes in population and the proportion of dependent people are associated with medical and long-term care expenditure.

van Baal et al. (16) examined the lifetime medical costs of obesity using a Markov model. They found that effective obesity prevention would reduce the cost of obesity-related diseases, but this reduction would be offset by the increased cost of diseases unrelated to obesity that would occur during the extra years of life gained. Although the present study did not directly consider obesity, the dependent state was partly caused by obesity-related diseases, such as stroke and musculoskeletal diseases, and thus similar findings to those of van Baal et al. (16) were obtained in a Japanese setting.

Several indicators of healthy life expectancy are used in Japan, including self-perceived healthy life expectancy and disability-free life expectancy (17). The number of people certified for long-term care needs is used to calculate disability-free life expectancy. There are two reasons why healthy life expectancy was not included in the results. First, as the present model used constant parameters for the death rates of independent and dependent people, it was difficult to precisely reproduce healthy life expectancy based on the reference data. Second, the present study used the number of long-term care recipients instead of the number of people certified for long-term care needs.

### Limitations

There are some limitations to this study. First, to hypothetically examine the effects of a reduction in the transition rate from the independent state to the dependent state on medical and long-term care expenditure, the model only analyzed the older Japanese population, and no feedback was obtained from the general working population. Second, the simulation model aimed to examine medical and long-term care expenditure as a whole and the change of disease structure was not considered. For a disease-specific simulation, the model should be modified. Third, for simplicity, constant parameters were set for death rates and transition rates from the independent state to the dependent state, as well as for per capita medical and long-term care expenditure. Therefore, these parameters remained the same across the simulated period. Further development of the model is necessary if the difference of these rates and expenditure needs to be considered by any factor. For example, by household structure, the transition rates from the independent state to the dependent state may be different as reported in Lin et al. (18) where the older Japanese living alone with dementia had a higher risk of care needs than those without dementia. Fourth, for simplicity, Level 2 to Level 5 of long-term care needs were combined to define the dependent state and to calculate long-term care expenditure. However, we believe that our simulation model, which combined the levels of long-term care needs separately within each sex and age group, was adequate for projecting healthcare costs and for comparing changes in these factors.

## Conclusions

Healthcare costs, including expenditure on medical care and long-term care, will increase as death rates decrease, but the increase in medical and long-term care expenditure will be attenuated by a reduction in the number of older people requiring long-term care.

## Data Availability Statement

The original contributions presented in the study are included in the article/supplementary material, further inquiries can be directed to the corresponding author/s.

## Author Contributions

NN designed the study, constructed the simulation model, and wrote the paper. NI and TS provided the critical revision of the model and the paper. KK and MM provided critical revision of the paper. All the authors approved the final version.

## Conflict of Interest

The authors declare that the research was conducted in the absence of any commercial or financial relationships that could be construed as a potential conflict of interest.
